# Coping in Pediatric Burn Survivors and Its Relation to Social Functioning and Self-Concept

**DOI:** 10.3389/fpsyg.2021.695369

**Published:** 2021-12-09

**Authors:** Mira D. H. Snider, Sarah Young, Paul T. Enlow, Corrine Ahrabi-Nejad, Ariel M. Aballay, Christina L. Duncan

**Affiliations:** ^1^Department of Psychology, West Virginia University, Morgantown, WV, United States; ^2^Department of Surgery, Burn Trauma Center, Western Pennsylvania Hospital, Allegheny Health Network, Pittsburgh, PA, United States; ^3^Center for Healthcare Delivery Science, Nemours Children’s Health System, Wilmington, DE, United States; ^4^Department of Pediatrics, Sidney Kimmel Medical College, Thomas Jefferson University, Philadelphia, PA, United States

**Keywords:** adaptive coping, pediatrics, burn survivors, social functioning, self-concept

## Abstract

Pediatric burn survivors experience increased risk for bullying, stigmatization, body image concerns, and problematic social functioning. Although coping behaviors are associated with engagement in social supports and positive self-concept in multiple pediatric illness populations, their relation has not been examined in pediatric burns. This study examined coping in relation to social functioning and self-concept in 51 pediatric burn survivors aged 7–17years (*M*=12.54; *SD*=2.65). Survivors and their caregivers completed the *Child Coping Strategies Checklist* (CCSC; youth report); the *Burn Injury Social Questionnaire* (BISQ; parent and youth report); and the *Piers-Harris Children’s Self-Concept Scale-2* (PH-2; youth report). Associations between coping, social functioning, self-concept, demographic features, and burn injury characteristics were examined *via* bivariate correlations. Hierarchical linear regressions examined whether coping strategies predicted social functioning and youth self-concept beyond burn injury and demographic variables. Social functioning concerns were positively correlated with total body surface area (TBSA; *r*=0.63 and 0.40, respectively). TBSA was the only significant predictor of parent-reported social concerns (*β*=0.65, *p*<0.001). Greater distraction coping predicted fewer youth-reported social concerns (*β*=−0.39, *p*=0.01). Greater active coping (*B*=0.67, *p*=0.002) and lower avoidance coping (*B*=−0.36, *p*=0.03) predicted better youth-reported self-concept. This study advances our understanding of coping as potentially protective for psychosocial adjustment. Clinicians working with child burn survivors should incorporate active coping interventions into treatment. Further research including larger and more diverse samples is needed to understand the role of coping approaches on psychological adjustment during burn healing.

## Introduction

Burns are among the leading causes of injury and unintentional death in the United States (American Burn Association, 2018). Pediatric populations are particularly vulnerable to burn injuries, with nearly a quarter of burn incidents in the United States occurring in children under 15years of age (American Burn Association, 2018). Although many burn injuries are not treated medically or can be treated in outpatient settings, severe injuries require hospitalization or transfer to a burn center for critical care (Krishnamoorthy et al., 2012). Treatment of burn injuries can include dressing changes, wound cleansing, medicated salve, pain management, skin grafting, physical and occupational therapies, and pressurized garments (Gill, 2010; Krishnamoorthy et al., 2012). Consequentially, burn care can be medically extensive, painful, and last months to years.

Along with their physical repercussions, burns are associated with impairments in psychosocial functioning that hinder healing, long-term adjustment, and overall well-being (Lavigne and Faier-Rouman, 1992; Pardo et al., 2008; Gill, 2010; Enlow et al., 2019). One domain of psychosocial functioning that can be specifically impacted by burns is social functioning. A systematic review of 75 articles focusing on the psychosocial outcomes of pediatric burn injuries revealed that survivors were at an increased risk for difficulties in social functioning several years post-burn (Bakker et al., 2013). Another study (Andersson et al., 2003) identified that parents of pediatric burn survivors perceived their children as having reduced social initiative, while teachers perceived students who survived burns as having a less prosocial orientation. Additionally, problems with peers were more common in children who had sustained burn injuries compared to the general population (Willebrand et al., 2011). Despite these findings, the extant literature on social adjustment in pediatric burns is not extensive, and this is an area in need of additional study.

Although not all burn survivors experience long-term psychosocial concerns (e.g., Koon, 1993; Bakker et al., 2013), the trauma of the injury itself, the pain of burn care, and potential physical outcomes (e.g., disfigurement, limited mobility) place youth at substantial risk for maladjustment (e.g., Gill, 2010; Kazis et al., 2017). It is critical for caregivers to support pediatric burn survivors such that they may engage in effective coping skills to ameliorate or prevent adverse psychological experiences (Meijer et al., 2002; Gill, 2010; Enlow et al., 2019). Compas et al. (2001) defines coping as “conscious, volitional efforts to regulate emotion, cognition, behavior, physiology, and the environment in response to stressful events or circumstances.” Specific psychosocial stressors that may be targeted by coping interventions in pediatric burn survivors include changes to social functioning and self-concept.

The physical sequelae from burns (e.g., visible scarring) can affect the self-concept of burn survivors, particularly during childhood and early adolescence, which is a formative period for a child’s sense of self (Koon, 1993). Youth who experience disfigurement from burn injuries have reported deficits in psychosocial functioning, including lower self-esteem, difficulties with self-concept and social skills, and higher levels of anxiety (Gill, 2010). A key factor that influences adjustment in pediatric burn survivors is the reaction of peers and those in public. Burn survivors are more likely to adjust poorly if peers react negatively toward their physical disfigurement (LeDoux et al., 1996), and survivors who experience debilitating physical symptoms may be limited in their social participation (Kazis et al., 2017). Conversely, psychosocial support from family and a trauma-informed healthcare team during the acute phase following a burn injury is important for promoting healthy adjustment (De Sousa, 2010; Bakker et al., 2013).

Coping behaviors are a significant predictor of self-esteem, social skills, social anxiety, and behavioral difficulties in chronically ill patients broadly (Meijer et al., 2002). Negative coping strategies, such as avoidance, have been associated with increased social difficulties in pediatric patients with neonatal brachial plexus injuries (Mentrikoski et al., 2015). Similarly, a cross-sectional study on youth with spinal cord injuries revealed an association between avoidant coping strategies, lower quality of life, and higher anxiety and depressive symptoms (Smith et al., 2013). In contrast, active coping strategies (i.e., seeking social support, problem solving, and openly talking about stressors) are most effective for promoting the psychosocial adjustment of chronically ill pediatric patients (Meijer et al., 2002) and a key to family functioning and support in pediatric oncology patients (Trask et al., 2003; Martin et al., 2012). In the context of pediatric burns, adaptive coping strategies are negatively associated with post-traumatic stress symptoms (Enlow et al., 2019), whereas internalizing (e.g., rumination, fixating on anxieties) and externalizing coping behaviors (e.g., screaming, breaking things) have been related to greater panic and general anxiety disorder symptoms (Rimmer et al., 2015).

Although there are several studies on the psychosocial impacts of pediatric burns, the relations among coping behaviors, social functioning, and self-concept are not well understood in this population. The current study expands upon extant research by addressing the following aims: (1) examine associations among coping behaviors, social functioning, self-concept, and burn injury-related variables; (2) investigate the extent to which coping strategies predict social functioning; and (3) assess the extent to which coping strategies predict self-concept in pediatric burn survivors.

## Materials and Methods

### Participants

Fifty-one pediatric burn survivors (*M* age=12.54; *SD*=2.65) and their primary caregivers participated. Families were recruited as part of a larger multi-project (e.g., Enlow et al., 2019) study examining the psychosocial outcomes of pediatric burn survivors. Eligible survivors were 7–17years of age and had sustained a burn requiring medical treatment at least 1month before recruitment. Survivors and caregivers were English speaking (measures validated only in English). Exclusion criteria included the child or caregiver having significant cognitive impairment, as reported by the primary caregiver or medical team that would preclude completion of study measures.

### Procedure

Researchers obtained hospital and university Institutional Review Board approval before project initiation. Participants were recruited during outpatient clinic appointments at two hospitals in Pennsylvania (*n*=14) and Ohio (*n*=4) and during registration for a summer camp in Pennsylvania for pediatric burn survivors (*n*=22). A recruitment letter and informed consent forms were mailed to burn survivors in the Pennsylvania hospital’s burn registry database who met inclusion criteria but were not approached in clinic or at camp (e.g., no longer required follow-up care), which yielded additional participants (*n*=11).

Caregivers and survivors recruited from clinic and camp each independently completed a questionnaire packet. Caregiver packets included the *Family Information Form* and *Burn Injury Social Questionnaire* (BISQ), while survivor packets included the *Child Coping Strategies Checklist* (CCSC), *BISQ*, and the *Piers-Harris Children’s Self-Concept Scale-2* (PH-2). Burn survivors recruited by letter from the hospital database were asked to contact research staff if interested in participating. They completed measures sent by mail, which were addressed individually for parent and child so that their responses could remain confidential from one another. Research staff also obtained data through medical records and the burn registry database.

### Measures

#### Family Information Form

The *Family Information Form* was designed for this study to obtain demographic information (i.e., gender, age, race, education, and socioeconomic status) from caregivers.

#### Chart Review Form

Research staff documented relevant medical history (i.e., burn characteristics, total body surface area of the burn; TBSA, associated surgeries and hospital stays, and scarring and disfigurement) for patients on the *Chart Review Form*.

#### Child Coping Strategies Checklist

The CCSC (Ayers et al., 1996) is a 52-item self-report questionnaire that measures coping behaviors over the past month when trying to solve problems. Items in this measure are rated on a 4-point Likert scale ranging from 1 (never) to 4 (most of the time), yielding four subscales: active coping, distraction strategies, avoidance strategies, and support seeking strategies. In our sample, internal consistency was high for the active coping (*α*=0.93) and support seeking subscales (*α*=0.88), good for the avoidance strategies subscale (*α*=0.75), and somewhat questionable for the distraction subscale (*α*=0.67).

#### Burn Injury Social Questionnaire

The BISQ is a brief, 10-item self-report screening questionnaire that was devised for this study to assess social functioning after a burn injury (e.g., “Kids tease me about the way I look”; “It is hard for me to have friends or dates because of my burn injury”). Items in this measure are rated on a four-point Likert scale ranging from 1 (not at all true) to 4 (very true). Parent- and child-report parallel versions are available; both versions have a Flesch-Kincaid grade level of below 1.0, suggesting that they are very easy to read and understand. Although this measure has not yet been validated, the items are face valid. Cronbach’s alpha for the current sample suggests good internal consistency for the BISQ-P (*α*=0.79) and BISQ-Y (*α*=0.72). A total score is calculated for analyses, with higher scores representing more problematic social concerns.

#### Piers-Harris Children’s Self-Concept Scale-2

The PH-2 (Piers and Herzberg, 2002) is a common, validated measure evaluating self-concept in youth. This 60-item questionnaire is rated using a yes/no format and is divided into six subscales: behavioral adjustment, intellectual and school status, physical appearance and attributes, freedom from anxiety, popularity, and happiness and satisfaction. The total standardized *t*-score for the PH-2 was used in analyses (*α*=0.89 for our sample); higher scores represent higher overall self-concept.

### Statistical Analysis

Bivariate correlations, independent samples *t*-tests, and ANOVA were used to examine the associations among coping behaviors, social functioning, self-concept, and demographic and burn injury-related variables (Aim 1). Hierarchical linear regression was used to assess whether coping strategies predicted social functioning (Aim 2) and self-concept (Aim 3). Demographic and burn injury-related variables significantly associated with self-concept and social functioning in bivariate analyses were included as covariates in the respective regression models.

## Results

### Demographics/Descriptive Statistics

Frequencies (Table 1) and descriptive statistics (Table 2) for demographic, clinical, and variables are reported for our sample. Due to the non-normal distribution of the family income data, participants were divided into quartiles (1st quartile=≤$29.9k, 2nd quartile=$30k–$49.9k, 3rd quartile=$50k–$89, and 4th quartile=≥$90k) for analyses. The median income was $40–$49.9k and the interquartile range was $60–$69.9k. The most common cause of burn was thermal (40.4%), followed by scald (32.7%). The average TBSA among participants was moderate (i.e., 9.19%), with approximately one-third having received skin graft surgery. Though the sample varied greatly with respect to time since injury (range=1–141.5months), participants completed study measures about 33months, on average, after their injury. Examination of PH-2 total scores revealed that approximately 4% (*n*=2) of participants had borderline low self-concept T-scores (≥1 SD but <2 SD below the mean) and around 2% (*n*=1) had significantly low self-concept T-scores (≥2 SD below the mean).

**Table 1:** Participant (*N*=52) demographics.

Variable	Value	*n* (%)
Gender	Male	33 (63.5%)
	Female	19 (36.5%)
Race	White	46 (88.5%)
	Non-White	6 (11.5%)
Yearly family income	<$10k	3 (5.8%)
	$10k–$19.9k	5 (9.6%)
	$20k–$29.9k	6 (11.5%)
	$30k–$39.9k	5 (9.6%)
	$40k–$49.9k	7 (13.5%)
	$50k–$59.9k	4 (7.7%)
	$60k–$69.9k	3 (5.8%)
	$70k–$79.9k	4 (7.7%)
	$80k–$89.9k	6 (11.5%)
	$90k–$99.9k	3 (5.8%)
	>$100k	5 (9.6%)
Cause of burn	Thermal	21 (40.4%)
	Scald	17 (32.7%)
	Contact	8 (15.4%)
	Electrical	1 (1.9%)
	Friction	1 (1.9%)
	Other	1 (1.9%)
Received skin graft	Yes	35 (32.7%)
Visible burn injury	Yes	46 (92.0%)

Thermal=burn caused by external heat source (e.g., flame); Scald=burn caused by contact with heated liquids (e.g., coffee); Contact=burn caused by contact with heated objects (e.g., hot iron); Electrical=burn caused by contact with an electric current (e.g., exposed wire); and Friction=burn caused by rubbing against another surface (e.g., treadmill).

**Table 2:** Descriptive statistics.

	*n*	Mean	SD	Min	Max
**Clinical variables**					
Time since burn (in months)	52	33.38	44.83	1.02	141.53
Total TBSA^a^	47	9.19	11.33	1.00	58.50
**Psychosocial variables**					
BISQ^b^ total (parent)	47	4.96	5.24	0.00	25.00
BISQ total (youth)	45	5.18	4.74	0.00	18.00
CCSC^c^ – active	46	2.65	0.65	1.25	3.96
CCSC – distraction	46	2.50	0.55	1.44	3.89
CCSC – avoidance	46	2.44	0.57	1.42	3.75
CCSC – support	46	2.31	0.78	1.00	3.56
PH-2^d^ total self-concept	48	47.77	9.79	11.00	60.00

a Total body surface area.

b Burn injury social questionnaire (BISQ).

c Child coping strategies checklist (CCSC).

d Piers-harris children’s self-concept scale – 2nd edition (PH-2).

### Bivariate Analyses

Results from bivariate correlations are presented in Table 3. To explore potential covariates (*p*<0.05) for regression models, we examined bivariate correlations of age, TBSA, and time since burn with each of our primary outcomes (BISQ and PH-2 total scores). TBSA was positively associated with parent report (*r*=0.63, *p*<0.001) and youth report on the BISQ (*r*=0.40, *p*=0.008). Youth report on the PH-2 was negatively associated with age (*r*=−0.34, *p*=0.04).

**Table 3:** Bivariate correlations.

	Age	Active coping	Distraction coping	Avoidance coping	Social support coping	BISQ – parent	BISQ – youth	PH2 – total	TBSA
Active coping	−0.10	---							
Distraction coping	0.04	0.35^*^	---						
Avoidance coping	−0.16	0.61^***^	0.27	---					
Social support coping	−0.14	0.66^***^	0.21	0.45^**^	---				
BISQ^a^ – parent	−0.20	0.03	−0.08	0.09	−0.01	---			
BISQ^a^ – youth	−0.01	−0.10	0.28	0.07	0.07	0.47^**^	---		
PH2^b^ – total	−0.34^*^	0.37^*^	0.24	−0.03	0.15	−0.17	−0.46^**^	---	
TBSA^c^	−0.07	0.01	0.10	0.04	0.04	0.63^***^	0.40^**^	0.08	---
Time since burn	0.17	0.06	0.17	0.08	0.10	0.08	−0.27	−0.02	0.09

* *p*<0.05;

** *p*<0.01;

*** *p*<0.001.

a Burn injury social questionnaire.

b Piers-harris children’s self-concept scale – 2nd edition.

c Total body surface area.

Correlations among primary study variables revealed that youth report on the PH-2 was negatively associated with youth reports on the BISQ (*r*=−0.42, *p*=0.005) and positively associated with youth use of active coping strategies (*r*=0.41, *p*=0.007). The use of active coping strategies was positively correlated with the use of distraction (*r*=0.35, *p*=0.017), avoidance (*r*=0.61, *p*<0.001), and social support coping strategies (*r*=0.66, *p*<0.001). The use of social support coping was also positively correlated with avoidance coping (*r*=0.45, *p*=0.002). As expected, BISQ-parent and BISQ-youth scores were positively correlated (*r*=0.47, *p*=0.001).

### Between-Group Comparisons

Independent-samples *t*-tests suggested that youth who had skin graft surgery reported more burn-related social problems on the BISQ (*M*=6.23, *SD*=5.28) than youth without a skin graft [*M*=2.86, *SD*=1.75; *t*(40.78)=−3.19, *p*=0.003; *d*=0.86]. There were no significant differences in youth PH-2 scores or parent BISQ scores between youth who did and did not have a skin graft. Male and female participants did not differ significantly in self-concept, or youth and parent report of burn-related social problems. Results from one-way ANOVA’s suggested BISQ and youth PH-2 scores were not statistically significant between family income quartiles (*p*’s>0.05).

### Regression Analyses

Hierarchical linear regression was used to examine whether coping strategies predicted (1) youth and (2) parent reports on the BISQ, as well as (3) youth report on the PH-2. Covariates (TBSA, presence of a skin graft) were entered in step one of the regression analysis, while all coping subscales from the CCSC were entered in step two. Results from three hierarchical linear regression analyses are reported in Table 4.

**Table 4:** Hierarchical linear regressions.

Predictor variables	Outcome measure					
	BISQ youth total		BISQ parent total		PH-2 total self-concept	
	*β*	Adj. *R*^2^	*β*	Adj. *R*^2^	*β*	Adj. *R*^2^
**Step 1**						
		0.17^*^		0.39^***^		0.04
Total TBSA	0.32^*^		0.63^***^		---	
Presence of graft	0.24		---		---	
Youth age	---		---		−0.25	
**Step 2**						
		0.30^*^		0.36		0.30^**^
Total TBSA	0.31^*^		0.65^***^		---	
Presence of graft	0.34^*^		---		---	
Youth age	---		---		−0.32^*^	
CCSC active	−0.25		0.09		0.67^**^	
CCSC distraction	−0.39^**^		−0.17		0.23	
CCSC avoidance	0.11		0.09		−0.36^*^	
CCSC support	0.28		−0.09		−0.18	
*F*(*df*)*^a^*	4.01 (6, 36)^**^		5.79 (5, 37)^***^		4.43 (5, 36)^**^	

a F-ratio for full model.

* *p*<0.05;

** *p*<0.01;

*** *p*<0.001.

The first model examined whether coping variables predicted youth reports on the BISQ. The full model was statistically significant [*F*(6, 36)=4.01, *p*=0.004] and accounted for 30% of the variance in youth reports of social problems. In the full model, greater use of distraction coping predicted fewer youth-reported burn-specific social problems (*β*=−0.39, *p*=0.01). Active, avoidance, and social support coping were not statistically significant predictors of youth reports on the BISQ. The presence of a skin graft (*β*=0.34, *p*=0.02) and TBSA (*β*=0.31, *p*=0.03) also significantly predicted more youth-reported burn-specific social problems on the BISQ.

The second model examined whether coping variables predicted parent reports on the BISQ. The full model was statistically significant [*F*(5, 37)=5.79, *p*<0.001] and accounted for 36% of the variance in parent reports of burn-specific social problems. In the full model, a greater TBSA predicted more parent reports of burn-specific social problems (*β*=0.65, *p*<0.001). None of the coping strategies were statistically significant predictors of parent reports on the BISQ.

The final model examined whether coping variables predicted youth self-concept as measured by the PH-2. The full model was statistically significant [*F*(5, 36)=4.43, *p*=0.003] and accounted for 30% of the variance in youth self-concept. In the full model, greater use of active coping strategies predicted better youth self-concept (*β*=0.67, *p*=0.002), while greater use of avoidance coping predicted worse youth self-concept (*β*=−0.36, *p*=0.03). Older youth also reported worse self-concept (*β*=−0.32, *p*=0.02). None of the other coping strategies were statistically significant predictors of youth reports on the PH-2.

## Discussion

Burn injuries in children can lead to substantial psychosocial concerns stemming from the trauma of the injury itself, its painful treatment, and its physical sequelae. Thus, pediatric burn survivors are at an increased risk of negative psychosocial outcomes such as anxiety, depression, acute stress disorder, PTSD, and deficits in self-concept and social functioning (Landolt et al., 2009; Bakker et al., 2013; Kazis et al., 2017). To date, there is limited research on how coping relates to self-concept and social functioning in these youth (LeDoux et al., 1996; Gill, 2010). This study investigated associations between coping behaviors, social functioning, self-concept, and individual characteristics in pediatric burn survivors, with a particular focus on whether specific types of coping predicted social functioning and self-concept.

Our first hypothesis that there would be associations between coping, social functioning, self-concept, and burn injury-related variables was partially supported. Lower youth self-concept was associated with more youth reports of burn-specific social problems and older survivor age. Higher self-concept was associated with greater use of active coping strategies. Youth and parent reports of more burn-specific social problems were also associated with higher TBSA.

Significant inter-correlations between coping subscales in our sample suggest that pediatric burn survivors rely on multiple forms of coping, rather than one specific or predominant coping strategy. This is similar to the findings of Camisasca et al. (2017), who found that youth who displayed secure attachment employed multiple coping strategies (i.e., active, social support, distraction, and avoidance) when faced with stressors. Likewise, Heffer and Willoughby (2017) alluded to the importance of coping flexibility with different coping strategies for different situations, which could explain why we obtained significant inter-correlations among coping strategies. The pattern of correlations was inconsistent, however, as not all coping subscales were correlated significantly with each other. It may be that inter-correlations were identified between active coping and social support, distraction, and avoidance coping because the use of active coping strategies involves attempts to reduce negative feelings and outcomes, which could consist of behaviors that resemble social support, distraction, and avoidance. Future research should continue to explore inter-correlations between use of different coping strategies to advance our understanding of the types of coping used in pediatric burn survivors and which combinations are most beneficial.

Our remaining hypotheses regarding the role of coping strategies as predictors of social functioning and self-concept were also partially supported. After controlling for burn injury variables and use of other coping strategies, greater use of distraction coping predicted fewer youth reports of burn-specific social problems. Similarly, less use of avoidance coping strategies and greater use of active coping strategies predicted lower youth self-concept after controlling for youth age and other coping strategies. Other coping strategies were not significant predictors of social functioning or self-concept in our sample. These findings align with prior research that suggests that the use of adaptive coping (e.g., active, social support, and distraction) is most effective in influencing positive youth psychosocial outcomes in youth with chronic illnesses (Meijer et al., 2002). Simultaneously, these results build upon the previous literature that found adaptive coping to be associated with positive parental mental health outcomes (Enlow et al., 2019). Adaptive coping is likely most effective because it requires youth to confront setbacks through problem solving and being cognizant of their thoughts and feelings toward the situation. Avoidance coping involves cognitive or behavioral strategies to avoid, deny, or minimize stressors and is associated with negative psychosocial outcomes (Smith et al., 2013; Mentrikoski et al., 2015). Consistent with past research, greater use of avoidance coping strategies predicted lower self-concept in pediatric burn survivors. However, use of avoidance coping was not significantly associated with burn-specific social problems in the current study. It may be that avoiding social problems with peers is less problematic and potentially adaptive in certain situations (e.g., staying away from bullies). Alternatively, this null finding may be due to the low statistical power of this study stemming from the small sample size.

Although the current study was conducted in the context of pediatric burns, our findings highlight the importance of adaptive coping strategies across illness populations. Empirically supported coping skills interventions can be delivered to help promote the use of adaptive coping strategies (e.g., open communication, conflict resolution, and problem solving; Jefferson et al., 2011). These interventions have been advantageous in other pediatric populations with chronic conditions, such as Type 1 diabetes (Hilliard and Hood, 2011). Taken together with the findings of the current study, it is likely that interventions which aim to increase the use of adaptive coping strategies (e.g., active, social support, and distraction), while decreasing the use of coping strategies associated with adverse psychosocial outcomes (e.g., avoidance coping) may be beneficial if tailored to pediatric burn survivors. In particular, a tailored intervention might focus on many of the same topics common to coping skills training (e.g., problem-solving, communication, and dealing with stress), but also target specific coping skills within burn-related scenarios (e.g., receiving wound care, responding to staring and bullying).

Consistent with prior research on burn-related psychosocial outcomes (Gill, 2010; Kazis et al., 2017), youth who had a skin graft stemming from a deeper burn and likely more scarring, reported more burn-related social problems. The association between burn-related variables and psychosocial outcomes has been mixed in the previous research. Some studies note that TBSA and scarring are associated with worse psychosocial outcomes (Karaçetin et al., 2014); yet, a meta-analysis concluded that clinical variables are not associated with psychosocial outcomes (e.g., anxiety, depression, and psychological maladjustment) in pediatric burn survivors (Noronha and Faust, 2007). Results from the current study suggest that burn-related variables may indeed be related to psychosocial outcomes and highlight a need for coping interventions to support survivors who experience more severe burn injuries. Active coping may underlie interventions that are often recommended for managing social reintegration after a severe burn (e.g., rehearsing responses in social situations, establishing contact with peer survivors; Phoenix Society, 2021). Although additional research is needed to fully understand the psychosocial impact of burn severity and skin grafts or other clinical variables in burn care, mental health interventions that emphasize coping in stressful social situations may be particularly beneficial for those survivors sustaining larger burn injuries, receiving a skin graft, or having visible scars or disfigurement.

A novel finding in the current study was the negative correlation between youth reports of burn-specific social problems and youth self-concept. It may be that youth who experience more social problems bear more negative feelings toward themselves. The previous research suggests that adolescents with negative self-concept are at a higher risk for social problems such as withdrawn behavior and internalizing problems (Ybrandt, 2008). With a larger sample, it would be possible to run analyses that examine the individual subscales of the PH-2 to have a clearer understanding of particular concerns in self-concept for pediatric burn survivors and how coping strategies foster or ameliorate these concerns. To this end, further evaluation of how specific coping approaches are related to specific aspects of self-concept may inform targeted mental health interventions.

### Limitations

There were several factors that may have limited the results of the current study. The relatively small sample size resulted in reduced statistical power in detecting small and medium effects. Moderate associations were identified between some specific coping strategies and youth report of burn-related social problems and youth self-concept; therefore, the reduced power may have contributed to the lack of statistically significant findings. Future research should build upon this study by recruiting larger samples to achieve better statistical power and accuracy of estimates. A wide range of time elapsed since burn injury also was observed in our sample, limiting the extent to which these results can be generalized to specific populations of burn survivors. Additionally, our sample was primarily White. This unintentional lack of racial and ethnic diversity in our sample further limits the generalizability of our findings. A more diverse sample would also help highlight cultural considerations. It should also be noted that for participants selected through the registry, respondents may not have been representative of the pool of participants to which those letters were sent. The authors were unable to draw conclusions about the generalizability of these findings to other registry members as this data was unavailable for analysis. Further, this study is cross-sectional and did not allow for an evaluation of psychosocial outcomes over time. Future research would be strengthened by a longitudinal design, which would allow for evaluations of trajectory changes in psychosocial adjustment across time. Additionally, future research may wish to examine how the association between coping and social functioning changes as a function of time since the burn injury.

### Strengths

The current study also demonstrated important strengths. For example, our sample was quite varied in terms of time since injury, which also was noted as a limitation. Our research design did not allow for examination of longitudinal trends within individuals but did permit a glimpse into how time since injury might related to psychosocial outcomes. An additional strength of this study was the use of both youth and parent reports of social functioning. This type of dual-report produced a more well-rounded understanding of each survivor’s social functioning and reduced shared-method variance that may have increased the odds of a Type I error. Given some advantages (i.e., readability, brevity) of the BISQ, it may be useful to adapt and validate this tool with other pediatric illness populations, especially those involving appearance-related concerns. Other tools, such as the Pediatric Quality of Life Inventory (Varni et al., 1999) and the Children’s Dermatology Life Quality Index (Parrish et al., 2020), include domains that measure interpersonal, social, and/or physical appearance concerns, which may be useful for validating the BISQ score. The inclusion of multiple coping strategies in relation to social functioning and self-concept was also a strength of this study, which is one of the first to measure the association between these particular psychosocial outcomes in pediatric burn survivors. As our understanding of coping develops, it would be beneficial to evaluate whether generic categories of coping that are studied across illness populations accurately reflect coping approaches used specifically in pediatric burns.

Observed associations between burn-injury variables, coping behaviors, social functioning and self-concept in this study suggest a need for psychosocial-based interventions that promote active coping and encourage further research of these specific outcomes in pediatric burn populations. Based on findings from previous studies (Holaday and McPhearson, 1997; Quezada et al., 2016), psychosocial interventions should focus on fostering positive social connections with family and peers as well as communication, social competence, problem-solving skills, self-esteem, and identification of self-worth in pediatric burn survivors. These factors are related to the development and growth of resilience – a characteristic that is essential for post-burn adjustment and well-being.

## Data Availability Statement

The raw data supporting the conclusions of this article will be made available by the authors, without undue reservation.

## Ethics Statement

The studies involving human participants were reviewed and approved by Allegheny Health Network Institutional Review Board and West Virginia University Institutional Review Board. Written informed consent to participate in this study was provided by the participants’ legal guardian/next of kin.

## Author Contributions

CD, PE, and MS conceived and devised study. PE contributed to data collection. SY, PE, CA-N, and MS were involved in statistical analysis. MS, SY, and PE contributed to writing. CD, CA-N, and AA were involved in manuscript editing. All authors contributed to the article and approved the submitted version.

## Funding

This research was supported by the West Virginia University Department of Psychology Student Research Fund.

## Conflict of Interest

The authors declare that the research was conducted in the absence of any commercial or financial relationships that could be construed as potential conflict of interest.

## Publisher’s Note

All claims expressed in this article are solely those of the authors and do not necessarily represent those of their affiliated organizations, or those of the publisher, the editors and the reviewers. Any product that may be evaluated in this article, or claim that may be made by its manufacturer, is not guaranteed or endorsed by the publisher.
